# Peri-Implant Crestal Bone Loss: A Putative Mechanism

**DOI:** 10.1155/2012/742439

**Published:** 2012-10-02

**Authors:** Yuko Ujiie, Reynaldo Todescan, John E. Davies

**Affiliations:** Institute of Biomaterials Biomedical Engineering and Faculty of Dentistry, University of Toronto, 164 College Street, Room 407, Toronto, ON, Canada M5S 3G9

## Abstract

*Purpose*. The immunological mechanisms of peri-implant crestal bone loss have, hitherto, not been elucidated. We hypothesized that bacterial products from the microgap cause upregulation of cytokines in otherwise healthy peri-implant cells, which results in osteoclast formation and, ultimately, in bone resorption. *Materials and Methods*. We used RT-PCR and ELISA to assay mediators of osteoclastogenesis in rat and human macrophages (r-and hMO); bone marrow derived stromal cells (r-and hBMCs); and human gingival fibroblasts (hGF)—with or without stimulation by LPS. TRAP positive multinucleate cells were assessed for their resorptive ability. *Results*. We show that IL-1**α**, IL-1**β**, and IL-6 were expressed by all examined cell types, and TNF-**α** was upregulated in hGF. Secretion of IL-1**α** and IL-1**β** proteins was stimulated in hMO by LPS, and IL-6 protein secretion was highly stimulated in hBMCs and hGF. Both LPS and RANKL stimulated macrophages to form osteoclast-like TRAP positive cells, which resorbed calcium phosphate substrates. *Conclusion*. Taken together, the results of our study support the hypothesis that bacterial endotoxins upregulate enhanced mediators of osteoclastogenesis in resident cells found in the healthy peri-implant compartment and that the local synergistic action of cytokines secreted by such cells results in the genesis of resorptively active osteoclasts.

## 1. Introduction

Dental implants facilitate the rehabilitation of edentulous patients, and markedly improve both function and esthetics. However, crestal bone loss on the order of 1.5 mm-1.6 mm has been observed radiographically around some dental implants within the first year of loading [1, 2]. The connection of the abutment to the endosseous component in two-stage systems invariably results in a microgap (10–50 *μ*m), which communicates with the much larger residual cavity created between the abutment screw and the internal implant wall. Previous evidence has shown that Gram-positive, and Gram-negative bacteria found in the oral cavity can colonize the inner surfaces of implants and the microgap area [3–5]; and cause inflammatory reactions in the peri-implant soft tissues. Furthermore, in a fascinating study Zipprich et al. [6] have shown that cyclical loading of the implant/abutment interface can result in the pumping of the liquid contained in implant cavities into the peri-implant compartment. 

The existence of an inflammatory cell infiltrate at the level of the implant-abutment junction—even around implants placed in areas of meticulous plaque control and clinically healthy, soft tissues—has been described in a series of animal studies [7–10]. However, other pre-clinical studies have reported a significant reduction in both inflammatory cell infiltrate and bone loss in both one-piece implants (compared to conventional two-piece implants) and designs in which the abutment/implant interface has been changed [9, 11]. Thus, it has been suggested that the microgap in two-piece implants plays a key role in the generation of an inflammatory cell infiltrate, and crestal bone loss, [12] through effects on hard and soft peri-implant tissues [13]. 

Broggini et al. demonstrated that in dogs, the major cell type constituting the inflammatory infiltrate around the microgap of submerged and nonsubmerged two-piece implants was the neutrophil [9]. In their experiment, while there was no selective neutrophil accumulation and a significant reduction of mononuclear cells adjacent to one-piece implants, the population of mononuclear cells was significantly increased around two-piece implants. Another study, in monkeys, showed an inflammatory cell infiltrate containing mostly lymphocytes and plasma cells in two-piece implants [14]. 

The pathophysiological consequences of the implant-abutment interface position have clinically important implications, since esthetic demands encourage the placement of implants in a more apical position [15]. Such placement could promote inflammation and bone loss, gingival recession, and esthetic failure. Relative to the original alveolar crest position, implants placed on the crestal or subcrestal level have demonstrated greater bone loss than implants placed supracrestally [13, 14, 16]. Additionally, experiments with subcrestal implant interfaces showed a significantly greater density of neutrophils than did supracrestal interfaces [10], but the size of the microgap was not a contributing factor in crestal bone resorption [12, 17]. A prospective clinical trial has identified a similar relationship between the location of the interface and the magnitude of bone loss [18], which ratifies the findings described above. In this clinical investigation, when the implant interface was positioned close to the original alveolar crest, greater bone loss occurred in comparison to implant interfaces placed more supracrestally. Thus, the location of the interface is an important determinant of alveolar bone loss in humans, as has been described in initial observations [19] and previous animal studies [20, 21]. 

It is widely known that bone matrix is resorbed by osteoclasts, which are multinucleated giant cells that originate from hematopoietic progenitors of the monocyte/macrophage lineage [22]. It has been reported that lipopolysaccharide (LPS) can induce bone resorption [23, 24] and osteoclast formation [25]. LPS supported the survival of preosteoclasts (mononuclear osteoclasts) through NF-*κ*B activation independent of host factors such as PGE_2_, RANKL, IL-1, and TNF-*α* [26]. Osteoclast formation can be modulated by IL-1*β* and TNF-*α* while enhancing the expression of RANKL [27]. LPS derived from different sources has been employed to specify an effector mechanism relevant to dental implants. Thus, while Tesmer et al. have shown that certain dental implant designs contribute to the development of multiple colony forming units for both *A. actinomycetemcomitans* and *P. gingivalis* [28], Koutouzis et al. [29] employed *E. coli* bacterial culture solution to demonstrate that implant design affects invasion of oral microorganisms into the fixture—abutment interface microgap under dynamic-loading conditions.

Thus, it has been shown that inflammatory cells, microbial cells and/or fluid [8–10, 14, 30] are associated with the implant-abutment interface. [10, 12, 14, 16] Nevertheless, the putative correlation between the microgap presence and crestal bone loss has remained refractory to mechanistic explanation. Consequently, the purpose of the present study was to investigate the possible mechanisms of crestal bone loss around dental implants using a bacterial endotoxin model *in vitro*. 

We hypothesized that bacterial products originating from the microgap cause the upregulation of cytokines, in healthy peri-implant macrophages, mesenchymal stromal cells and gingival fibroblasts that result in the recruitment of resorptively active osteoclasts and, ultimately, bone resorption. The notion that healthy fibroblasts in the periodontium could play an important role in providing sustainable inflammatory mediators in the transition from gingivitis to periodontitis has also recently been discussed [31], although such cells would not be expected in the peri-implant compartment. Thus, in order to address our question, we started with the premise that the peri-implant tissues surrounding the microgap are normal and healthy at the time of healing abutment connection. We further assumed that bacterial products would act on cells in the supra-bony soft tissue, and thus we investigated the effect of LPS on cytokine expression and secretion in macrophages, stromal cells, and gingival fibroblasts. To qualify our LPS model, we first undertook some comparative studies to assess the effects of laboratory produced, *Porphyromonas gingivalis* (*P*. *gingivalis*), *Prevotella intermedia* (*P*. *intermedia*), and commercially available, *Escherichia coli* (*E*. *coli*), LPS on human gingival fibroblasts, since concern has recently been raised over the use of “home made” LPs preparations [32].

## 2. Material and Methods

### 2.1. Preparation of Human Gingival Fibroblasts (hGFs) from Gingival Tissue

Samples of gingival tissues were obtained, with informed consent, from 5 patients who showed no clinical signs of periodontal disease. The tissues were collected from different sites: (1) the palate, used for free connective tissue gingival grafting; (2) the marginal gingivae, following extraction of third molars; (3) the gingiva around dental implants during 2nd stage surgery. The collected tissue was washed 3 times with *α*-MEM supplemented with Penicillin G (167 units/mL), Gentamicin (50 *μ*g/mL), and 10% FBS, following which it was cut into small pieces and digested with a mixture of 3 mg/mL collagenase type 1 (Sigma-Aldrich) and 4 mg/mL Trypsin in SM for 1 hour at 37 degrees. The digested tissue was centrifuged for 5 minutes at 1150 rpm, and the supernatant was discarded. Then, 10 mL of SM was added to the tube containing the pellet, and the cell suspension was transferred to a T-75 culture flask. When cells reached 80–90% confluence, they were passaged to new T-75 flasks and plated at a concentration of 1.0 × 10^6^ cells/cm^2^ in 6-well plates at Passage 3. This procedure was repeated five times.

### 2.2. Human Macrophages (hMO)

Two healthy volunteers, a 35-year-old female and a 50-year-old male, donated blood for this experiment, after providing informed consent. Venous blood (30 mL) was collected from a brachial vein into heparinized tubes and immediately processed. Peripheral blood mononuclear cells (PBMC) were isolated using gradient centrifugation in Histopaque solution (Sigma-Aldrich). Isolated PBMCs were incubated in RPMI (Roswell Park Memorial Institute) medium-1640 (Gibco) with 10% FBS in 6 well-plates at a density of approximately 5 × 10^6 cells/cm^2^ in a final volume of 2 mL. The cells were allowed to adhere to plastic dishes for 90 minutes at 37 degrees, 5% CO_2_, in RPMI medium. The cells were vigorously washed with PBS three times, and the nonadherent cells (mainly lymphocytes) were removed. Cell viability was 91% before cells were seeded and characterized by immunofluorescence analysis with Human anti-CD14, Human MHC II, and FITC-conjugated Mouse IgG (all from BD Scientific).

#### 2.2.1. Human Bone Marrow Cells (hBMCs)

hBMCs were obtained, following local Research Ethics Board (REB) approval, from the collection kit filters (Baxter, Deerfield, IL, USA) normally discarded following harvesting of bone marrow, from healthy donors, for transplantation. The cells were washed with PBS and then plated in 75 cm^2^ flasks in 10 mL of SM. Cultures were incubated at 37 degrees with 5% CO_2_. After 24 hours, non-adherent cells were removed. When cells achieved 70–80% confluency, they were trypsinized (0.25% trypsin in PBS at 37 degrees for 5 minutes), harvested, and expanded in new 75 cm^2^ flasks. Cells were expanded to achieve 80–85% confluency at Passage 1 through Passage 4. At this stage, no hemopoietic lineage cells were detectable. The hBMCs were suspended in SM and seeded at a concentration of 1.0 × 10^6^ cells/cm^2^ in 6-well plates at Passage 4. We collected filters from six different donor aspirates and repeated the hBMCs culture procedures six times.

### 2.3. Mouse Macrophages (RAW 264.7) (mMO)

The mouse macrophage cell line, RAW 264.7 (ATCC, Manassas, VA, USA), was initially suspended in fresh supplemented medium (SM) composed of 10% fetal bovine serum (FBS) (Hyclone, Logan, UT, USA), 80% DMEM (ATCC) and 10% antibiotics (167 units/mL Penicillin G, 50 *μ*g/mL Gentamicin), and 0.3 *μ*g/mL Fungizone (all Sigma-Aldrich, St. Louis, MO, USA). Subsequently, the cells were plated in 75 cm^2^ (T-75) polystyrene tissue culture flasks (BD, Franklin Lakes, NJ, USA), and incubated at 37 degrees with 5% CO_2_. The medium was replaced every 2 or 3 days. The cells grew to 80% confluency, as observed by inverted phase microscopy, after which they were washed with PBS (Gibco, Carlsbad, CA, USA) and removed from the culture flask after the addition of 2.5 mL 0.25% trypsin (Gibco) in PBS for 5 minutes at 37 degrees. To inactivate the trypsin, 5 mL SM was added, and the cell suspension was then centrifuged at 1150 rpm for 5 minutes. The total viable cell count was obtained from a ViCell-XR Automated Cell Counter (Beckman Coulter, Fullerton, CA, USA). This cell culture protocol was followed for cell expansion from passage 0 to passage 2. On passage 3, RAW 264.7 cells were plated at a concentration of 2.0 × 10^5^ cells/well in 6-well plates. These cell culture procedures were repeated 6 times.

### 2.4. Rat Stromal Bone Marrow Cells (rSBMCs)

rSBMCs were harvested from the femora of 100–120 g Wistar rats. The harvested femora were soaked and washed for 10 minutes three times with *α*-MEM medium (Gibco) supplemented with 10% antibiotics (as above). Both epiphyses were cut off and the bone marrow was gently flushed out from both sides with a 10 mL syringe (fitted with a 20-gauge needle) filled with fully supplemented medium (FSM: 10% FBS, 80% *α*-MEM and 10% antibiotics). The volume of bone marrow suspension was adjusted to 30 mL with FSM and divided in two aliquots of 15 mL each, which were distributed in two T-75 flasks. The first medium change was done at 24 hours and then three times per week subsequently. Cells were grown for 5 to 6 days to 80–85% confluence. At each passage, and up to passage 8, 1.0 × 10^6 cells were collected and fixed with formalin in a 1.5 mL microcentrifuge tube for flow cytometry (Beckman Coulter, Miami, FL, USA) analysis of the CD45 positive cell population (CD45, mouse anti-rat Caltag Laboratories, Burlingame, CA, USA), for investigation of the presence of hematopoietic cells. Aliquots of 2.0 × 10^6 cells/cm^2^ of the rSBMCs at passage 7 were seeded into 6-well plates. The passage 7 was chosen in this assay because passage 7 included only 0.36% of CD45^+^ cells. Stromal cells were isolated from bone marrow of two rats for quantification of CD45^+^ cells.

### 2.5. LPS Preparations

hGFs were stimulated with different types of LPS: *P*. *gingivalis*, *P*. *intermedia*, and *E*. *coli LPS* (*Escherichia coli* 055:B5, Sigma-Aldrich). Both LPS of *P*. *gingivals* and *P*. *intermedia* were isolated from colonies of these bacterial cultures. The strains used in this study were *P*. *intermedia* ATCC 25611, *P*. *gingivalis* ATCC 33277. All anaerobes were maintained on Brucella HK agar (Kyokuto, Tokyo, Japan) supplemented with 5% laked sheep blood in an atmosphere of 80% N_2_, 10% CO_2_, and 10% H_2_O in the aerobic chamber for 48 hours 37 degrees. LPS was extracted by using an LPS extraction kit (iNtRON Biotechnology, Kyunggi-do, Korea), dissolved in 10 mM Tris-HCl buffer, and freeze-dried.

### 2.6. LPS Stimulation of Cells

In separate experiments, hBMCs and hGFs were stimulated with *E*. *coli* LPS (0 *μ*g/mL or 0.1 *μ*g/mL or 1 *μ*g/mL) for 24, 48, or 96 hours, and they were stimulated with *P. gingivalis* LPS or *P. intermedia* LPS (1 *μ*g/mL) for 2 or 24 hours (37 degrees, 5% CO_2_).

RAW 264.7 cells were stimulated with *E. coli* LPS (*Escherichia coli *055:B5, Sigma-Aldrich) (0 *μ*g/mL or 1 *μ*g/mL) for 12, 24, 36, 48, and 60 hours; hMO were stimulated with LPS (0 *μ*g/mL or 1 *μ*g/mL) for 24 hours; rSBMCs were stimulated with *E. coli* LPS (0 *μ*g/mL or 0.1 *μ*g/mL or 1 *μ*g/mL) for 6, 24, 48 and 96 hours; hBMCs and hGFs were stimulated with *E. coli* LPS (0 *μ*g/mL or 0.1 *μ*g/mL or 1 *μ*g/mL) for 24, 48, and 96 hours. In each case, LPS was added to the culture medium when cells reached 75–80% confluence, which was approximately at day 3 for mMO and day 7 for rSBMCs, hGFs, and hBMCs. LPS was added into the culture medium every 24 hours. Cell culture supernatants of hBMCs and hGFs were harvested and stored at −80 degrees prior to enzyme-linked immunosorbent assay (ELISA, Thermo Fisher Scientific, Waltham, MA, USA). RNA was isolated from both LPS stimulated cells and cell, lysate after the culture medium was removed (see below).

### 2.7. Detection of Cytokine Expression (RT-PCR)

Total cellular RNA was extracted from hBMCs, hGFs,, hMO, mMO and rSBMCs. with 1 mL/10 cm^2^ culture dish area of TRI Reagent (Ambion, Austin, TX, USA). cDNA templates of these cells were produced using a reverse transcriptase kit (Quanti Tect Reverse Transcription kit, Qiagen, Mississauga, ON, USA). GAPDH, a house-keeping gene, was used for all species of human, rat, and mouse. Specific primers of IL-1*α*, IL-1*β*, TNF-*α*, IL-6, RANK, RANKL, TRL-2, TRL-4, CD14 and MD-2 were designed for human or rat, and mouse individually using an electronic gene database (*BLAST, National Library of Medicine*). The specific primers (Table 1) were added to each cDNA sample to be amplified using a Taq polymerase kit (Platinum Taq DNA polymerase, Invitrogen, Carisbad, CA, USA). Expression of cDNA was assessed by running 25 to 35 cycles at 55 degrees to 65 degrees on reverse transcriptase-polymerase chain reaction (RT-PCR) amplification. Amplified products were analyzed by 1.5% agarose gel electrophoresis and visualized with ethidium bromide (Sigma-Aldrich) staining. RT-PCR was repeated at least 3 times for each cell type. 

### 2.8. Determination of Cytokine Secretion (ELISA)

Culture supernatants from each cell type were removed and stored at −80 degrees prior to ELISA analysis. ELISA kits were used to quantify IL-1*α*, IL-1*β*, TNF-*α*, and IL-6 concentration in cell-free culture supernatants according to the manufacturer's protocols. hBMCs and hGFs were extracted with a lysis buffer (Cell Signaling Technology, Beverly, MS, USA) and the protein lysates were stored in the same manner. ELISA kits were used to quantify IL-1*α*, IL-1*β*, and TNF-*α* concentration in cell lysates according to the manufacturer's protocols. The absorbance at 450 nm was read with wavelength correction set at 550 nm. The sensitivities of the commercial ELISA kits were <2 pg/mL for IL-1*α*, <1 pg/mL for IL-1*β*, <2 pg/mL for TNF-*α*, and <1 pg/mL for IL-6. The number of samples of cell-free culture supernatant samples, the cell lysate samples, and the times of repeated analysis of each sample are shown in Table 2. The concentration of IL-1*α*, IL-1*β*, IL-6, and TNF-*α* was analyzed in cell-free culture supernatants and the cell lysate for hBMCs and hGFs. In hMO, we analyzed only the concentration of IL-1*α* and IL-1*β*. 

### 2.9. TRAP (Tartrate Resistant Acid Phosphatase) Staining

mMO (RAW 264.7) were washed with PBS and fixed with 10% formalin at room temperature for 5 minutes. Then, they were washed with distilled water and incubated in michaelis veronal acetate buffer (pH 5.0) containing naphtol AS-BI phosphate as substrate, pararosanilin-HCL as coupler, and tartaric acid for detection of TRAP activity, for 40 minutes at 37 degrees. After removal of the TRAP incubation buffer, cells were washed with distilled water. They were analyzed and counted in micrographs taken with a digital camera attached to the light microscope. 

### 2.10. TRAP Assay

mMO cells in a 96-well plate were washed with PBS and add 100 *μ*L/well of the assay solution: 1 ml of 0.1 M pNPP (disodium p-Nitrophenylphophate hexahydrate), 1 ml of ×10 buffer (1 M sodium acetate and 0.1 M sodium tartrate), 1 ml of 0.1% Triton-X, and fill up to 10 ml with distilled water. Incubate the plate for 10 minutes at room temperature and add 50 *μ*L/well of 0.2 M NaOH as a stop solution. Then, measure the absorbance at 405 nm.

### 2.11. Bone Resorption Assay

The osteoclastic bone resorption assay was performed using the commercially available OSTEOLOGIC culture system (BioCoat Osteologic Bone Cell Culture System, BD), in which the substrate is coated with a thin layer of osteoclast resorbable calcium phosphate ceramic [33]. RAW 264.7 cells (1.0 × 10^4 cells/well) were cultured on the OSTEOLOGIC plates for 1 day. Then, LPS (1 *μ*g/mL), or RANKL (receptor activator of nuclear factor *κ*B ligand) (200 ng/mL) was added to the cell culture medium, and cells were stimulated with either LPS or RANKL for 7 days. Un-stimulated cells were used as controls. After 7 days, the cell culture medium was removed and cells were fixed with 10% formalin. Micrographs of the OSTEOLOGIC plates were taken with SEM (Scanning electron microscopy). This cell culture procedure was repeated three times. 

## 3. Results

### 3.1. Effects of Various LPS on Pro-Inflammatory and Osteoclastogenic Gene Expression

We investigated the effects of different types of LPS on pro-inflammatory and osteoclastogenic cytokine gene expression following exposure to LPS for 2 hours in hGF. 

**Figure 1:**
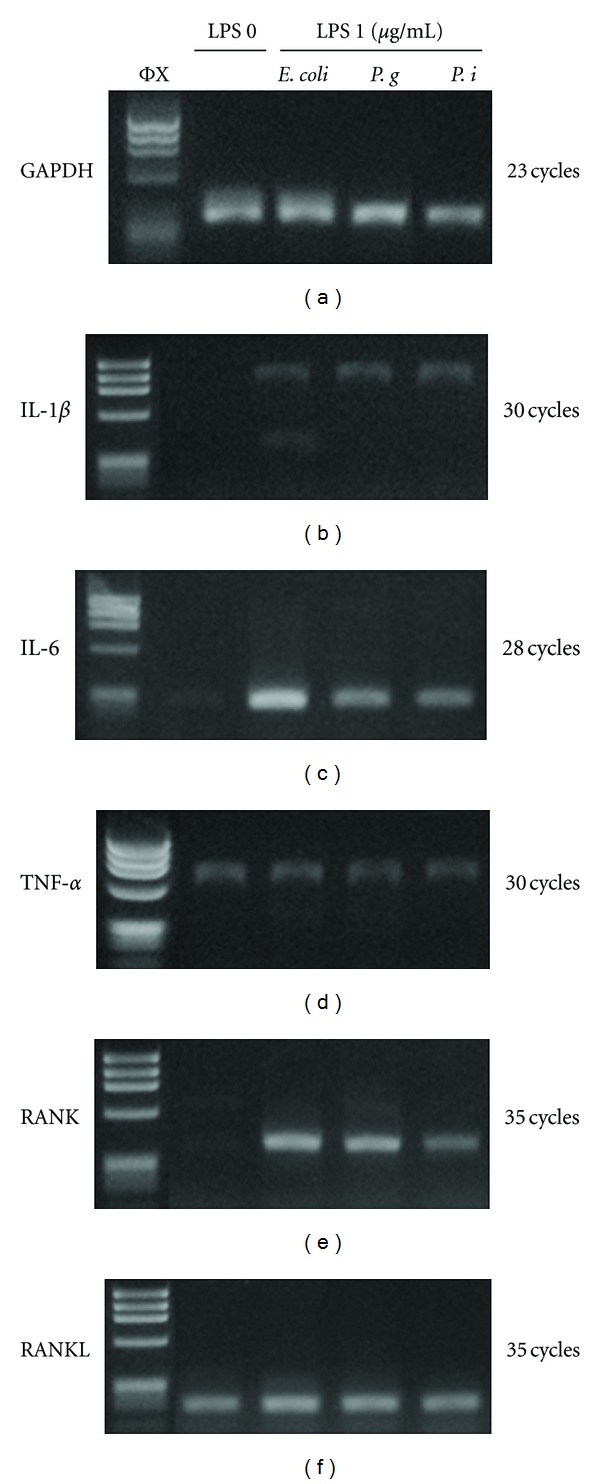
Expression of pro-inflammatory and osteoclastogenic genes with several LPS stimulation in human gingival fibroblasts. IL-1*β*, IL-6, TNF-*α*, RANL, RANKL, and GAPDH gene expression in hGF cultures with different types of LPS stimulation: *E*. *coli*, *P*. *gingivalis*, *P*. *intermedia* and without LPS for 2 hours. 1 *μ*g/mL of LPS was used in the experiment. mRNA was isolated at 2 hours. RT-PCR was carried out at different cycles for each gene. Annealing temperature was 55 degrees for all genes.

All three LPS types (derived from *E.coli*, *P. gingivalis*, *P. intermedia*.) showed similar upregulation of IL-1*β*, IL-6, and RANKL gene expression (Figures 1(b), 1(c), and 1(e)). TNF-*α* and RANKL expression was detected in both non-LPS and LPS conditions (Figures 1(d) and 1(f)).

### 3.2. Effects of Different LPS on LPS Receptors and Their Related Gene Expression

**Figure 2:**
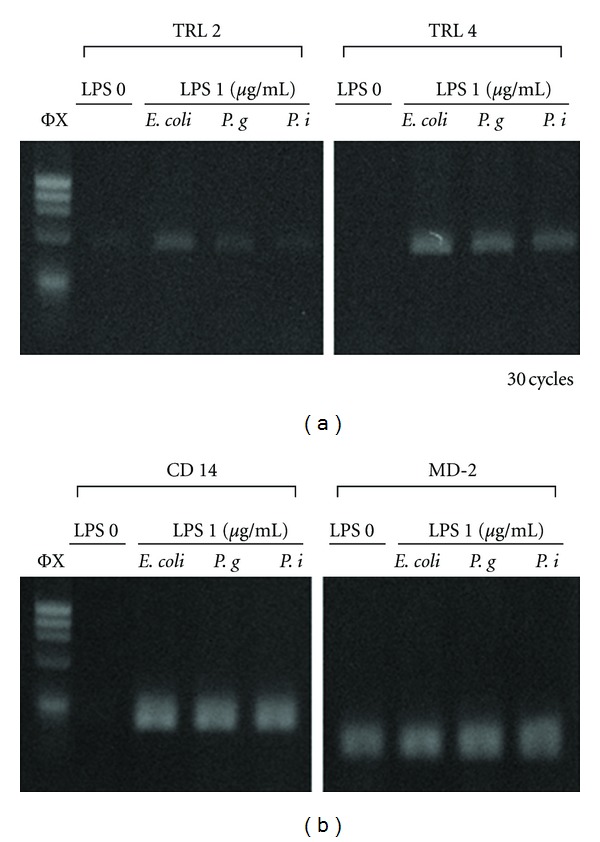
Expression of LPS receptors and their signaling factor's genes with several LPS stimulation in human fibroblasts. TRL 2, TRL 4, CD14, and MD-2 gene expression in hGF cultures with different types of LPS stimulation: *E. coli*, *P. gingivalis*, *P. intermedia,* and without LPS for 2 hours. 1 *μ*g/mL of LPS was used in the experiment. mRNA was isolated at 2 hours. RT-PCR was carried out at different annealing temperature that TRL2 and CD14 were 57 degrees, and TRL 4 and MD-2 were 54 degrees. PCR cycle was 30 cycles for all genes.

We next examined the effects of LPS on the TRL 2 and TRL 4 LPS receptors and their signaling cascade factors (Figure 2(a)). Without LPS, no TRL 2 or TRL 4 gene expression was observed. This was also the case for CD-14 gene expression, although MD-2 was expressed with or without LPS stimulation (Figure 2(b)). 

These assays illustrated that all three LPS extracts exhibited similar effects. Therefore, for convenience, we selected *E. coli *LPS for subsequent experiments as it is commercially available.

The examination of various LPS effect on gene expression was analyzed using hGF. However, our experimental purpose was to investigate the cellular influence on osteoclastogenesis in the surrounding tissue cells. Therefore, we also examined the effect of macrophages and bone marrow cells, which could be expected to exist around dental implant fixtures, from human, mouse, and rat.

### 3.3. Human Macrophages

**Figure 3:**
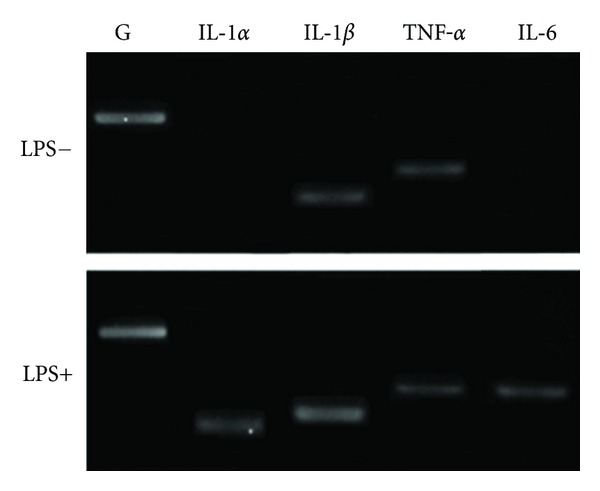
Expression of pro-inflammatory cytokine genes in human macrophages. IL-1*α*, IL-1*β*, TNF-*α*, IL-6, and GAPDH gene expression in hMO cultures with and without LPS stimulation for 24 hours. 1 *μ*g/mL of LPS was used in the experiment. mRNA was isolated at 24 hours. RT-PCR was carried out at 30 cycles for all genes.

hMO expressed IL-1*β* and TNF-*α* gene in the presence or absence of LPS for 24 hours at 35 cycles of RT-PCR (Figure 3). Both IL-1*α* and IL-6 expression was increased by LPS stimulation in human macrophages at 30 cycles of RT-PCR (Figure 3). 

### 3.4. Mouse Macrophages

**Figure 4:**
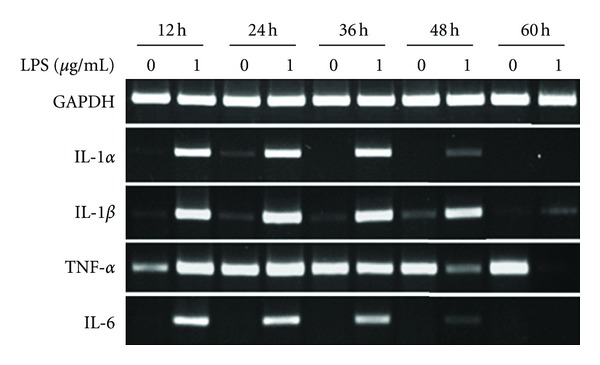
Expression of pro-inflammatory cytokine genes in mouse macrophages (RAW 264.7). Gene expression of IL-1*α*, IL-1*β*, TNF-*α*, IL-6, and GAPDH in mMO cultures in the absence (0 *μ*g/mL) and presence (1 *μ*g/mL) of LPS. RNA was isolated at 12, 24, 36, 48, and 60 hours. RT-PCR was carried out at 30 cycles for all genes in this experiment.

mMO cultures showed that the expression of IL-1*α*, IL-1*β*, and IL-6 genes was upregulated with LPS stimulation at 30 cycles of RT-PCR (Figure 4). However, gene expression of TNF-*α* was observed irrespective of LPS stimulation at all time points, except for 60 hours (Figure 4). Therefore in our experiments, TNF-*α* expression was not upregulated by LPS. The IL-1*α* and IL-6 gene expression decreased at 48 hours, while IL-1*β* gene expression was decreased at 60 hours after LPS stimulation (Figure 4).

### 3.5. Human Bone Marrow Cells

**Figure 5:**
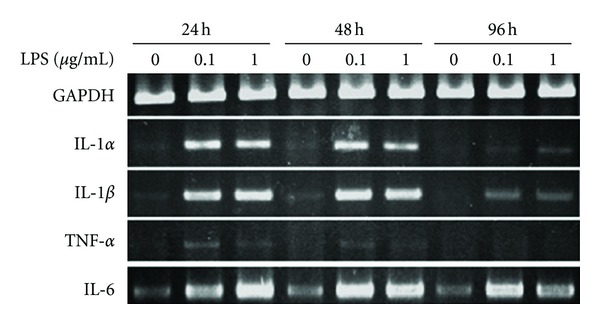
Expression of pro-inflammatory cytokine genes in human bone marrow cells. IL-1*α*, IL-1*β*, TNF-*α*, IL-6, and GAPDH gene expression in hBMCs cultures with or without LPS stimulation. LPS concentrations were 0 *μ*g/mL, 0.1 *μ*g/mL or 1 *μ*g/mL. RNA was isolated at 24, 48, and 96 hours. RT-PCR was carried out at 30 cycles for GAPDH, IL-1*α*, IL-1*β*, and TNF-*α*, and 25 cycles for IL-6.

hBMCs showed a similar trend for gene expression of cytokines as that found with rSBMCs (Figure 5). The gene expression of IL-1*α*, IL-1*β*, and IL-6 was stimulated by the presence of LPS. However, the expression of IL-1*α* and IL-1*β* gene was reduced at 96 hours in hBMCs culture. TNF-*α* gene expression also occurred in the presence of LPS at 24 hours and 48 hours (Figure 5). Nevertheless, the TNF-*α* expression was very weak at each LPS concentration and time point. The LPS-stimulated genes for IL-1*α* and IL-1*β* were observed by RT-PCR at 30 cycles, and for IL-6 gene at 25 cycles. 

### 3.6. Rat Stromal Bone Marrow Cells

**Figure 6:**
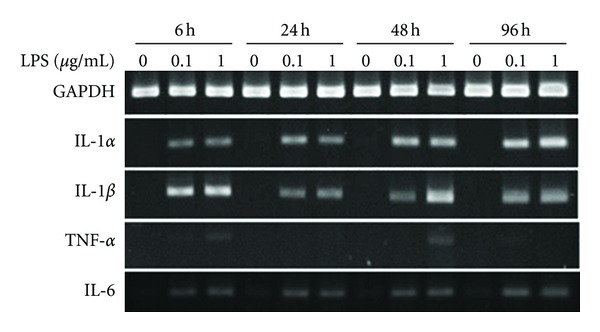
Expression of pro-inflammatory cytokine genes in rat stromal bone marrow cells. Gene expression for IL-1*α*, IL-1*β*, TNF-*α*, IL-6 and GAPDH in rSBMCs cultures in the presence and absence of LPS. The LPS concentrations used were 0.1 *μ*g/mL and 1 *μ*g/mL. RNA was isolated at 6, 24, 48 and 96 hours. RT-PCR was carried out at 30 cycles for GAPDH, IL-1*α*, IL-1*β*, and TNF-*α*, and 25 cycles for IL-6.

LPS upregulated gene expression of IL-1*α*, IL-1*β*, and IL-6 during the rSBMCs poststimulation period (6–96 hours, Figure 6), showing the same trend of mMO cultures. The expression of TNF-*α* gene in the presence of LPS at 6 and 48 hours at 30 cycles was very weak (Figure 6). Running cycles for RT-PCR were same as hBMCs.

Although we used hGF for estimating the effect of different LPS in this study, those experiments were carried out with 2 hours LPS exposure. We also investigated the LPS effects against hGF with longer time periods, 24, 48, and 96 hours, in a subsequent experiment. 

### 3.7. Human Gingival Fibroblasts

**Figure 7:**
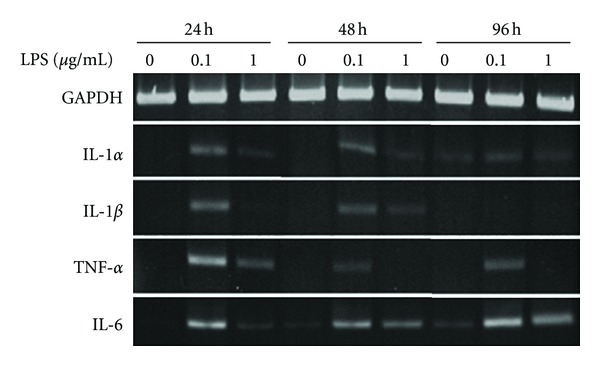
Expression of pro-inflammatory cytokine genes in human gingival fibroblasts. Gene expression for IL-1*α*, IL-1*β*, TNF-*α*, IL-6, and GAPDH genes in hGF cultures with and without LPS stimulation. LPS concentrations were 0 *μ*g/mL, 0.1 *μ*g/mL or 1 *μ*g/mL. RNA was isolated at 24, 48, and 96 hours. RT-PCR was carried out at 30 cycles for GAPDH, 35 cycles for IL-1*α*, IL-1*β*, and TNF-*α*, and 25 cycles for IL-6.

All genes, for IL-1*α*, IL-1*β*, TNF-*α*, and IL-6, were expressed in the presence of LPS at all time points in hGF cultures. However, expression of IL-1*β* was not expressed at 96 hours in spite of increased (+5) cycles; the expression of IL-1*α*, IL-1*β*, and TNF-*α* was observed at 35 cycles. Interestingly, all gene expression was stronger at 0.1 *μ*g/mL than at 1 g/mL LPS (Figure 7). LPS-stimulated IL-1*α* and IL-1*β* gene expression was reduced in a time-dependent manner, however, IL-6 gene expression increased especially with 1 *μ*g/mL concentration of LPS (Figure 7). The expression of IL-6 gene was observed at 25 cycles. 

## 4. Effect of LPS on Protein Secretion

ELISA was used to assess levels of protein secretion (IL-1*α*, IL-1*β*, TNF-*α*, and IL-6) in cultures of hMOs, hBMCs, and hGFs. 

### 4.1. Human Macrophages

Significant secretion of IL-1*α* and IL-1*β* was found in the cell-free culture supernatant after stimulation with LPS for 24 hours (Table 3(a)). IL-1*α* and IL-1*β* concentrations were approximately 470 times and 12.5 times higher in the presence of LPS. 

### 4.2. Human Bone Marrow Cells

The analysis of cell lysate by ELISA revealed that LPS-stimulated hBMCs secreted IL-1*α* at 24 hours and IL-1*β* at 24, 48, and 96 hours (Table 3(b)) but these protein levels were very low. Secretions of TNF-*α* from protein lysate were detected both with and without LPS at all time points (Table 3(b)). However, the levels of TNF-*α* secretion were also very low. Thus, the secretion of IL-1*α*, IL-1*β*, and TNF-*α* was meaningless in hBMCs cultures. 

**Figure 8:**
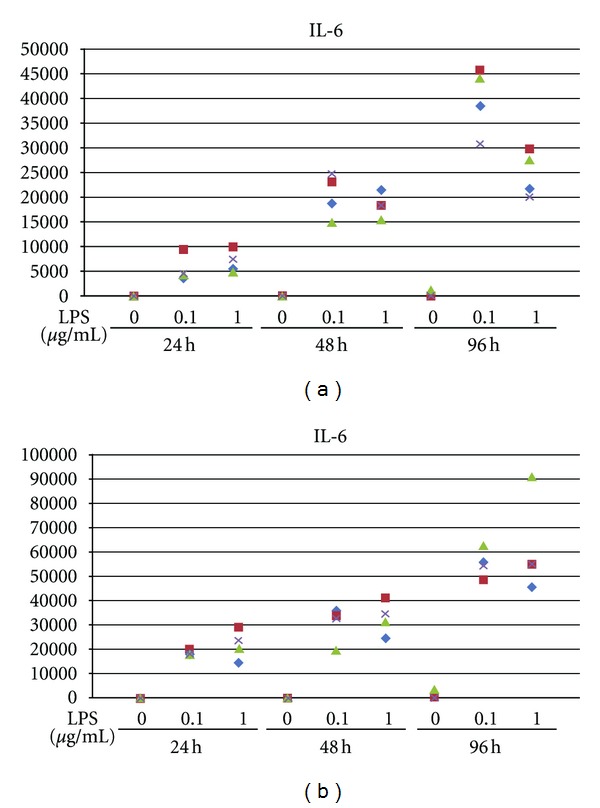
(a) Secretion of IL-6 protein in human bone marrow cells IL-6 secretion in hBMCs cultures with and without LPS stimulation for 24, 48, and 96 hours. LPS concentrations were 0 *μ*g/mL, 0.1 *μ*g/mL or 1 *μ*g/mL. The amount of IL-6 in cell-free culture supernatants was analyzed by ELISA. (b) secretion of IL-6 protein in human gingival fibroblasts Secretion of IL-6 in hGFs with or without LPS stimulation for 24, 48, and 96 hours. LPS concentrations were 0 *μ*g/mL, 0.1 *μ*g/mL or 1 *μ*g/mL. The amount of IL-6 in cell-free culture supernatants was analyzed by ELISA.

To the contrary, the level of IL-6 secretion was extremely high in the presence of LPS in hBMCs (Figure 8(a)). IL-6 concentration was approximately 40000 times higher at 96 hours in the presence of LPS as maximum compared to absence of LPS. The secretions of IL-6 from cell-free culture supernatants were detected from only with LPS at all time points (Figure 8(a)). Therefore, the secretion of IL-6 protein was induced by LPS stimulation in a time-dependent manner, but not an LPS dose-dependent manner. 

### 4.3. Human Gingival Fibroblasts

Secretion of TNF-*α* was not specific to the presence of LPS stimulation at 24, 48, and 96 hours (Table 3(c)). The secretion of TNF-*α* was not induced by LPS stimulation in hGFs. 

The levels of IL-6 protein were clearly increased with LPS in hGFs (Figure 8(b)). The secretions of IL-6 were detected from cell-free culture supernatants at all time points: 24, 48 and 96 hours. Secretion of IL-6 by hGFs in the presence of LPS stimulation was found to be time- and dose-dependent hGFs (Figure 8(b)).

## 5. Effect of LPS on the Differentiation of Mouse Macrophages into Osteoclasts

### 5.1. Observation of Differentiation of Osteoclasts

#### 5.1.1. TRAP Assay

**Figure 9:**
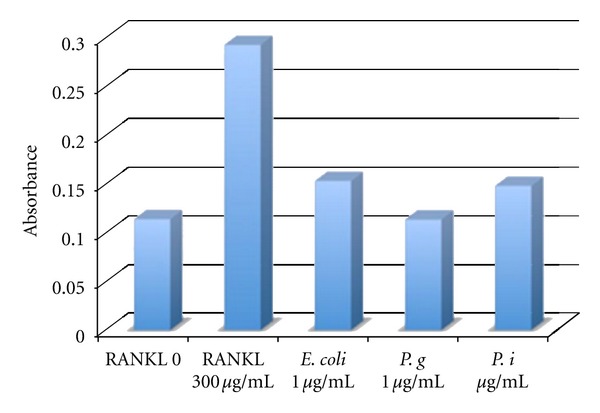
TRAP positive reactions were showed as level of absorbance in each group. The data were calculated by triplicate samples in each group. One day after mMO cells were seeded into 96 wells they were stimulated with *E. coli*, *P. gingivalis*, *P. intermedia* LPS for 3 days. 1 *μ*g/mL of LPS was added into the cell culture medium individually. As positive control, we used 300 ng/mL GST-RANKL which was treated the same way with LPS groups. Negative control was not including RANKL and LPS.

mMO (RAW 264.7 cells) were cultured in medium with 300 ng/mL GST-RANKL for 3 days demonstrated the highest TRAP positive reaction compared to negative controls, without RANKL, and LPS stimulated mMO (Figure 9). The various LPS did not show different TRAP positive ability. The various LPS did not show different TRAP positive ability mostly. 

#### 5.1.2. TRAP Staining

**Figure 10:**
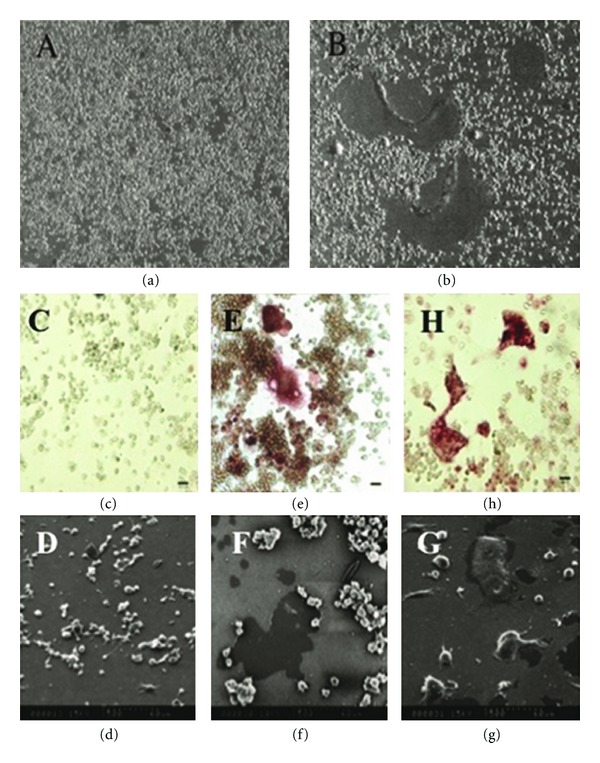
(a) and (b) Cell morphology of mouse macrophages in the presence of LPS Cells images were taken by phase contrast microscope (original magnification, ×10). mMO (RAW 264.7) were cultured for 7days in the absence of LPS (a) and in the presence of 1 *μ*g/mL LPS (b). (c) to (g) Osteoclast-like cells in RAW264.7 cultures induced by LPS and RANKL mMO (RAW 264.7) were incubated with 1 *μ*g/mL of LPS or 200 ng/mL of RANKL for 7 days. TRAP staining images were taken by phase contrast microscope (c, e and h). Pit formation activity of osteoclasts was observed by SEM (d, f and g). (c and d) mMO seeded on CaP substrate (control), (e and f) treated with LPS and (h and g) treated with RANKL.

mMO were cultured in medium containing 1 *μ*g/mL LPS for 5 to 7 days and observed under phase contrast for the presence of multinucleated cells (MNCs) (Figure 10(b)). The MNCs were enlarged the size, but the amount of MNCs was decreased at day 7. mMO did not show morphological changes in the absence of LPS (Figure 10(a)).

Osteoclast formation was examined by TRAP staining, which is a selectable staining to identify osteoclasts and activated macrophages. TRAP-positive mononuclear cells and MNCs were observed in mMO cultures in the presence of LPS (Figure 10(e)) and in the presence of RANKL (Figure 10(h)). mMO cultures without the addition of LPS or RANKL were used as negative control and did not show positive reaction to TRAP staining (Figure 10(c)). mMO cultures stimulated with RANKL were used as a positive control (Figure 10(h)).

### 5.2. Observation of Activation of Osteoclasts

Pit forming activity was analyzed using the commercially available BioCoat Osteologic Bone Cell Culture System (Osteologic), which consists of quartz plates with a thin film surface coating of calcium phosphate (CaP). Pit formation on the CaP substrates was observed in mouse macrophage cultures in the presence of LPS (Figure 10(f)) and RANKL (Figure 10(g)). Control cultures did not show any pit formation (Figure 10(d)).

## 6. Discussion

Although gingival and periodontal pathologies are known to cause the upregulation and secretion of pro-inflammatory cytokines, [34–36] our studies were aimed at elucidating a mechanism whereby dental implants, placed in healthy tissue, and could cause the genesis of osteoclasts that would be the effectors of peri-implant crestal bone loss. Since the latter is a local, not systemic phenomenon, there is a need to understand the local causative mechanisms. Thus, we hypothesized that bacterial products, originating from bacteria colonizing implant/abutment connections, could cause the upregulation of pro-inflammatory cytokines in the cells of healthy peri-implant tissues that would, in turn, stimulate the recruitment of resorptively active osteoclasts. Indeed, our results show that *E. Coli* LPS, as a model bacterial endotoxin, can selectively stimulate the upregulation of pro-inflammatory cytokines in healthy candidate resident cells, which can cause the genesis of osteoclasts that are resorptively active. 

Despite differences in implant/abutment designs, implant/abutment connections will invariably form a microgap, that under function (cyclic opening/closing), will allow passage of organic fluids containing bacteria and endotoxin from and to the implant cavities [7]. Furthermore, the bacterial colonization of the internal cavities of two piece dental implants has been repeatedly demonstrated through both microscopical observations and *in vitro* studies [3–5]. In fact, it is likely that these cavities provide anaerobic chambers for pathogens that could induce an immune response in the host [9]. The intensity of such response would depend on: the immunologic profile of each individual (as is known to be the case in periodontal disease [37]); the amount and types of periodontal pathogens present in the mouth; possibly, the position (in relation to the bone crest) of the implant/abutment interface [11, 13]. Indeed, there seems to be a delicate balance between cytokines that trigger immunological response (pro-inflammatory) and those that will modulate the immunological response [38]. Interestingly, clinical studies of implant/abutment self-locking connections [39, 40], which are more stable and reduce fluid percolation (with respect to the less stable flat-to-flat nonself-locking components) [7], have shown a reduction in the overall loss of crestal bone. 

Our results show that LPS upregulated IL-1*α* and IL-6 gene expression in all the examined cells types; while IL-*β*, and TNF-*α* were only upregulated in cultures of hBMCs, and hGFs. On the contrary, analysis of protein secretion showed very low or undetectable levels IL-1*α*, IL-1*β* and TNF-*α* in cultures of hM, hBMCs and hGFs. However, there was a clear increase in IL-6 secretion, with LPS treatment, for both hBMCs and hGFs, a finding that corresponds to previous reports [41, 42]. Indeed, IL-6 has been shown to stimulate osteoclast activity through enhancement of the expression of RANKL with soluble IL-6 receptors (sIL-6R) in both calvarial bones [43] and osteoblasts [44], an effect reliant on the synergistic effect of IL-6 and sIL-6R [45]. Furthermore, IL-6 can stimulate osteoclast-like formation in long-term human bone marrow cultures by inducing IL-1*β* release [46]. Thus, IL-6 might be responsible for osteoclast differentiation and activation through enhancement of RANKL expression [43–45]. 

IL-6 has also been implicated to have a role on the pathogenesis of periodontitis. Gingival tissues obtained from diseased periodontal sites contain higher levels of IL-6 as compared to healthy sites [47] and crevicular fluid from periodontally diseased sites contain more IL-6 than healthy sites [48]. While IL-*β*, IL-10, IL-12, IL-8, IL-6, and TNF-*α* have all been found to be present in peri-implant crevicular fluid, recorded levels tended to be higher than those observed around teeth, [41] and IL-6 expression is stimulated by IL-1*α* and TNF-*α* in hGFs [42]. Osteoblasts have also been shown to produce IL-6 in response to local bone-resorbing agents [49]. Thus, it is likely that IL-6, in cooperation with other cytokines, plays a role in the bone [36] destruction and tissue damage. In fact, increases in inflammatory cytokines are associated with bone loss and progressive failure of dental implants [50, 51]. Since cytokines are small molecules of short life span and short acting distance—which can produce responses in very small concentrations and in a paracrine fashion—[52] this may explain why the recent introduction of platform switching in implant design [53, 54] contains the pro-inflammatory cytokine production to the area between the abutment and platform; thereby shielding the surrounding bone from their deleterious effects.

In comparison to the specific expression and abundant secretion of IL-6 by hBMCs and hGFs, it is noteworthy that low amounts of IL-1*α*, IL-1*β*, and TNF-*α* were also produced and secreted. The IL-1*α* and IL-1*β* have been shown to enhance RANKL expression in both osteoblasts and stromal cells [44, 45], while IL-1*α* promotes the survival of osteoclasts through NF-kB activation which results in induction of osteoclast activation [44]. These findings suggest that IL-1*α* induces differentiation and activation of multinucleate osteoclasts [55]. TNF-*α* stimulates the differentiation and survival of osteoclasts, but not the function of osteoclasts [56]. Thus, the differentiation and activation of osteoclasts could be stimulated by LPS through IL-1*α*, IL-1*β*, and TNF-*α*. Our results show that LPS activated macrophages can form resorptively active osteoclast-like TRAP positive cells and suggest that LPS may have a direct action on resident macrophages in addition to its known ability to act indirectly on preosteoclasts [23]. 

## 7. Conclusion

Taken together, our results support the hypothesis that bacterial endotoxins may upregulate pro-inflammatory genes in a number of resident cells found in the healthy peri-implant compartment, and, that the local synergistic action of cytokines secreted by such cells result in the genesis of resorptively active osteoclasts.

## Figures and Tables

**Table:** (a) Specific primers for mouse

Specificity	Primer	Detection	Product size (bp)
IL-1alpha	Sense	TGCCACCAAAGAACAAAGTCG	599
	Antisense	CCCACTGAGGTAGGAAAGATGTAGC	

IL-1beta	Sense	TCTTTGAAGTTGACGGACCCC	269
	Antisense	CCAGCAGGTTATCATCATCATCCC	

TNF-alpha	Sense	ACCGTCAGCCGATTTGCTATC	288
	Antisense	TCAGAGTAAAGGGGTCAGAGTGGG	

IL-6	Sense	GAGCCCACCAAGAACGATAG	229
	Antisense	TCCACGATTTCCCAGAGAAC	

**Table:** (b) Specific primers for rat

Specificity	Primer	Detection	Product size (bp)
IL-1alpha	Sense	AGGTTCCACGTTTCCTCCTT	233
	Antisense	GCCTCCAGGTCATCTTCAGT	

IL-1beta	Sense	CAACAAAAATGCCTCGTGC	395
	Antisense	AAGTCAACTATGTCCCGAC	

TNF-alpha	Sense	TACTGAACTTCGGGGTGATCG	292
	Antisense	CCTTGTCCCTTGAAGAGAACC	

IL-6	Sense	TGTGCAATGGCAATTCTGAT	156
	Antisense	GGAACTCCAGAAGACCAGAGC	

**Table:** (c) Specific primers for human

Specificity	Primer	Detection	Product size (bp)
IL-1alpha	Sense	TTCATTGGCGTTTGAGTCAG	163
	Antisense	GGAGTGGGCCATAGCTTACA	

IL-1beta	Sense	GCATCCAGCTACGAATCTCC	193
	Antisense	TCGTTATCCCATGTGTCGAA	

TNF-alpha	Sense	ACAAGCCTGTAGCCCATGTT	265
	Antisense	TTGATGGCAGAGAGGAGGTT	

IL-6	Sense	GCTATGAACTCCTTCTCCACAAGC	264
	Antisense	TTCTGCCAGTGCCTCTTTGC	

RANK	Sense	ATGCGGTTTGCAGTTCTTCT	384
	Antisense	CGTAGGGACCACCTCCTACA	

RANKL	Sense	AGAGCGCAGATGGATCCTAA	180
	Antisense	TTCCTTTTGCACAGCTCCTT	

TRL 2	Sense	TTAGCAACAGTGACCTACAGAG	503
	Antisense	CAAATCAGTATCTCGCAGTTCC	

TRL 4	Sense	TGGATACGTTTCCTTATAAG	507
	Antisense	GAAATGGAGGCACCCCTTC	

CD 14	Sense	CAACTTCTCCGAACCTCAGC	271
	Antisense	TAGGTCCTCGAGCGTCAGTT	

MD-2	Sense	GCACATTTTCTACATTCC	157
	Antisense	CACAGTCTCTCCCTTCAG	

**Table:** (d) Specific primers for House keeping gene

Specificity	Primer	Detection	Product size (bp)
GAPDH	Sense	ACCACAGTCCATGCCATCAC	452
	Antisense	TCCACCACCCTGTTGCTGTA	

**Table 2:** The samples for ELISA analysis were cell-free culture supernatants and protein lysate. Both the cell-free culture supernatant samples and the protein lysate samples of hBMCs and hGFs were obtained from three different donors and analyzed twice. The cell-free culture supernatant samples of hMO were obtained from one donor. The LPS without samples were analyzed twice and the LPS with samples were analyzed four times. The protein lysate samples were not prepared from hMO.

The samples for ELISA	Human Macrophages		HBMCs		HGFs	
Cell-free culture supernatant	LPS −	LPS +	LPS −	LPS +	LPS −	LPS +
	*n* = 1	*n* = 1	*n* = 3	*n* = 3	*n* = 3	*n* = 3
	Analyzed twice	Analyzed four times	Analyzed twice	Analyzed twice	Analyzed twice	Analyzed twice

Protein lysate	LPS −	LPS +	LPS −	LPS +	LPS −	LPS +
	×	×	*n* = 3	*n* = 3	*n* = 3	*n* = 3
	×	×	Analyzed once	Analyzed once	Analyzed once	Analyzed once

**Table:** (a) Secretion of IL-1*α* and IL-1*β* by human macrophages after LPS stimulation

	IL-1*α*		IL-1*β*	
Stimulation period	LPS 0	LPS 1 *μ*g/mL	LPS 0	LPS 1 *μ*g/mL
24 hrs	0 pg/mL	470.80 ± 79.69 pg/mL	32 pg/mL	400 pg/mL

**Table:** (b) Secretion of IL-1*α*, IL-1*β*, and TNF-by human bone marrow cells after LPS stimulation

	IL-1*α* (pg/mL)			IL-1*β* (pg/mL)			TNF-*α* (pg/mL)		
	LPS (*μ*g/mL)			LPS (*μ*g/mL)			LPS (*μ*g/mL)		
Stimulation period	0	0.1	1	0	0.1	1	0	0.1	1
24 hrs	0	5.49 ± 1.21	5.11 ± 2.03	0	9.17 ± 4.57	16.15 ± 1.78	19.32 ± 10.03	52.95 ± 5.26	54.55 ± 3.25
48 hrs	0	0.34 ± 0.46	0	0	0	15.59 ± 1.05	22.27 ± 2.12	51.48 ± 2.33	40.00 ± 4.82
96 hrs	0	0	0	0	0	0	14.89 ± 2.44	42.39 ± 8.85	40.11 ± 11.06

**Table:** (c) Secretion of IL-1*α*, IL-1*β*, and TNF-by human gingival fibroblasts after LPS stimulation

	IL-1*α* (pg/mL)			IL-1*β* (pg/mL)			TNF-*α* (pg/mL)		
	LPS (*μ*g/mL)			LPS (*μ*g/mL)			LPS (*μ*g/mL)		
Stimulation period	0	0.1	1	0	0.1	1	0	0.1	1
24 hrs	0	0	0	0	0	0	23.30 ± 3.72	28.30 ± 1.14	26.93 ± 2.18
48 hrs	0	0	0	0	0	0	22.27 ± 2.05	17.84 ± 8.17	21.36 ± 3.20
96 hrs	0	0	0	0	0	0	20.00 ± 4.16	19.31 ± 6.89	22.84 ± 1.76
